# Understanding Cell Lines, Patient-Derived Xenograft and Genetically Engineered Mouse Models Used to Study Cutaneous T-Cell Lymphoma

**DOI:** 10.3390/cells11040593

**Published:** 2022-02-09

**Authors:** Raman Preet Kaur Gill, Jennifer Gantchev, Amelia Martínez Villarreal, Brandon Ramchatesingh, Elena Netchiporouk, Oleg E. Akilov, Niels Ødum, Robert Gniadecki, Sergei B. Koralov, Ivan V. Litvinov

**Affiliations:** 1 Division of Dermatology, McGill University, Montreal, QC H4A 3J1, Canada; raman.gill@mail.mcgill.ca (R.P.K.G.); jennifer.theoret@mail.mcgill.ca (J.G.); amelia.martinezvillarreal@mail.mcgill.ca (A.M.V.); brandon.ramchatesingh@mail.mcgill.ca (B.R.); elena.netchiporouk@mcgill.ca (E.N.); 2 Department of Dermatology, University of Pittsburgh, Pittsburgh, PA 15213, USA; akilovoe@upmc.edu; 3 Division of Dermatology, University of Alberta, Edmonton, AB T6G 2B7, Canada; ndum@sund.ku.dk; 4 Skin Immunology Research Center, University of Copenhagen, DK-2200 Copenhagen, Denmark; r.gniadecki@ualberta.ca; 5 Department of Pathology, New York University, New York, NY 10016, USA; sergei.koralov@nyulangone.org

**Keywords:** cutaneous T-cell lymphoma, adult T-cell leukemia/lymphoma, CD30, mycosis fungoides, sézary syndrome, chromosomal aberration, expression profiling, TP53, TOX, TAX, xenograft mouse model, genetically engineered mouse models, HTLV-1

## Abstract

Cutaneous T cell lymphoma (CTCL) is a spectrum of lymphoproliferative disorders caused by the infiltration of malignant T cells into the skin. The most common variants of CTCL include mycosis fungoides (MF), Sézary syndrome (SS) and CD30^+^ Lymphoproliferative disorders (CD30^+^ LPDs). CD30^+^ LPDs include primary cutaneous anaplastic large cell lymphoma (pcALCL), lymphomatoid papulosis (LyP) and borderline CD30^+^ LPD. The frequency of MF, SS and CD30^+^ LPDs is ~40–50%, <5% and ~10–25%, respectively. Despite recent advances, CTCL remains challenging to diagnose. The mechanism of CTCL carcinogenesis still remains to be fully elucidated. Hence, experiments in patient-derived cell lines and xenografts/genetically engineered mouse models (GEMMs) are critical to advance our understanding of disease pathogenesis. To enable this, understanding the intricacies and limitations of each individual model system is highly important. Presently, 11 immortalized patient-derived cell lines and different xenograft/GEMMs are being used to study the pathogenesis of CTCL and evaluate the therapeutic efficacy of various treatment modalities prior to clinical trials. Gene expression studies, and the karyotyping analyses of cell lines demonstrated that the molecular profile of SeAx, Sez4, SZ4, H9 and Hut78 is consistent with SS origin; MyLa and HH resemble the molecular profile of advanced MF, while Mac2A and PB2B represent CD30^+^ LPDs. Molecular analysis of the other two frequently used Human T-Cell Lymphotropic Virus-1 (HTLV-1)^+^ cell lines, MJ and Hut102, were found to have characteristics of Adult T-cell Leukemia/Lymphoma (ATLL). Studies in mouse models demonstrated that xenograft tumors could be grown using MyLa, HH, H9, Hut78, PB2B and SZ4 cells in NSG (NOD Scid gamma mouse) mice, while several additional experimental GEMMs were established to study the pathogenesis, effect of drugs and inflammatory cytokines in CTCL. The current review summarizes cell lines and xenograft/GEMMs used to study and understand the etiology and heterogeneity of CTCL.

## 1. Introduction

Cutaneous T cell lymphomas (CTCLs) are a heterogeneous group of non-Hodgkin’s lymphomas characterized by the origin of malignant T cells in the skin [1,2]. The most diagnosed variants include mycosis fungoides (MF), Sézary syndrome (SS), and primary cutaneous anaplastic large cell lymphoma (pcALCL). These three variants together account for ~70% of all CTCL cases [3]. Previous studies suggested that MF/SS most often develops in Caucasians >55 years of age. In African-American, Hispanic and Middle-Eastern individuals disease develops at around 40–45 years of age [4,5,6,7]. A hypopigmented variant is commonly seen in children, adolescents, young adults, predominantly in individuals with Fitzpatrick phototype IV-VI skin [8].

MF is a lymphoma of the skin, but may also affect lymph nodes, blood, bone marrow, and rarely visceral organs. Conventional Alibert-Bazin MF is typically characterized by erythematous patches and plaques on the trunk following a bathing suit distribution. However, as the disease progresses, nodules/tumors can develop. These tumors may present with large cell transformation, an enlargement of clonal lymphoma cells into phenotypically large cells, which is associated with poor prognosis. The confluent skin involvement (erythroderma) is another evidence of advanced disease. At advanced stages, malignancy can involve lymph nodes, be detected in blood, and can progress metastasizing into the visceral organs. Furthermore, patients affected by any form of CTCL are at risk of developing additional lymphomas [9] or in rare cases (in cytotoxic types of CTCL) have serious complications including hemophagocytic lymphohistiocytosis (HLH) [10].

SS, an aggressive leukemic variant of CTCL, is characterized by a triad of erythroderma, enlarged lymph nodes, and the leukemic level of malignant T cells with convoluted/cerebriform nuclei in the blood [11].

Another common subtype(s) of CTCL are the CD30^+^ lymphoproliferative disorders (CD30^+^ LPDs), that account for ~10–25% of CTCL and include 3 conditions: lymphomatoid papulosis (LyP), borderline CD30^+^ LPD, and pcALCL. CD30^+^ LPDs usually have overall a favorable prognosis, but a morbid clinical course requiring treatment. These patients are at the risk of developing other lymphoid malignancies. LyP is characterized by recurring dome-shaped papulonecrotic or nodular skin lesions occurring in cropped or generalized eruptions, often presenting on the trunk and proximal extremities. These lesions spontaneously regress after a few weeks; involution with crusting, ulceration, and possible scarring. pcALCL is characterized by solitary or grouped nodules or tumors that are rarely multi-focal and show common cutaneous relapse with possible spontaneous regression in some patients [12]. The 5 year disease-free survival is 95% and 99% in pcALCL and LyP patients, respectively [13]. Additional details on variants of MF/CTCL have been extensively reviewed elsewhere [14,15].

CTCL can be often misdiagnosed as benign inflammatory skin conditions such as chronic eczema and psoriasis [16]. On average, it takes ~6 years to establish this diagnosis [17]. As mentioned above, while only a small proportion of CTCL patients progresses to advanced stages, despite recent research progress, it still remains unpredictable which patients will progress. To better understand the disease etiology, improve diagnosis/prognosis and develop novel therapies, it is important to understand the existing CTCL research models and strive to develop new ones.

Immortalized patient-derived cancer cell lines are being used to study genomics, proteomics, and molecular mechanisms involved in disease pathogenesis. They are critical for drug screens to identify compounds with anticancer activity. In addition, these patient-derived cell lines along with primary patient cells are used in xenograft/GEMMs to investigate and validate the translational research findings and to test therapeutic interventions in settings that enable scrutiny of not just an impact on primary tumor cells but also on the tumor microenvironment (TME) in the skin.

The current review summarizes the origin of the immortalized CTCL cell lines, defines variants of CTCL they belong to, and summarizes the genomic and proteomic studies carried in these models. In addition, this review provides details on existing xenograft/GEMMs available to study the development and progression of the disease and highlights their potential use to validate and optimize therapies prior to human studies.

## 2. Patient-Derived Cell Lines

### 2.1. Karyotype Analysis and Chromosomal Aberrations

Patient-derived immortalized cancer cell lines, albeit hard to establish, are readily available, easy to use and provide primary platforms for evaluating molecular pathways associated with malignant transformation and identification of cancer cell intrinsic vulnerabilities for drug screening purposes. Currently, 11 immortalized patient-derived cell lines are used to study CTCL (Table 1) [18,19,20]. CTCL cells have been studied extensively with the aim to explore their genomic and transcriptomic similarities among cell lines (as a group) as well as between cell lines and patient samples [21,22,23,24,25,26].

Netchiporouk et al., (2017) studied the CTCL cell lines by using spectral karyotyping. Specifically, 5 cells of each cell line were studied to identify chromosomal alterations in available CTCL cell lines. It was noted that different chromosomes were altered in each cancer T-cell, highlighting the molecular heterogeneity of the disease. The study also revealed that cell lines representing MF, CD30^+^ LPDs, and SS exhibit significant and persistent genomic instability, suggesting that many of the tumor lines used in the field are readily evolving with each passage [36]. The most common observation in MyLa (advanced MF), PB2B (CD30^+^ LPDs), Mac2A (CD30^+^ LPDs), HH (advanced leukemic MF), Hut78 (SS), H9 (SS), SeAx (SS), Sez4 (SS) and SZ4 (SS) cells was significant aneuploidy. The notable exception was two HTLV-1^+^ cell lines MJ and Hut102, which had very little indication of genomic aberrations and/or aneuploidy. These two cell lines were diploid and exhibited very few structural alterations. These cells strongly expressed the TAX HTLV-1 oncogene. These differences highlight that while Hut102 and MJ were derived from patients with erythematous plaques (Hut102 derived from lymph node and MJ derived from blood), they likely represent ATLL and not MF/SS.

The spectral karyotyping analysis followed by gene expression analysis of these cells revealed the presence of similar alterations across the different cell lines. Hut78 and H9 were found to carry several unique and many shared chromosomal alterations indicating the common origin of these cell lines, as expected [36]. Karyotype and gene expression analyses confirmed that PB2B and Mac2A cell lines carry the same number and type of chromosomal alterations/gene expression profile changes supporting the fact that they represent similar advanced clinical disease (from the same patient) at different stages [36,37,38]. Spectral karyotyping analysis and gene expression profiling of Sez4 and SZ4 cells revealed that these are nearly identical cell lines as they exhibited similar chromosomal alterations but acquired different names in literature [36].

### 2.2. Comparison of Molecular Markers in Cell Lines and Patient Samples

The highlighted studies based on karyotyping of cell lines [21,22,23,24,25,39,40,41,42,43,44,45,46,47,48] were correlated with chromosomal aberrations seen in patients. SS and MF patient studies demonstrated similar chromosomal gains and losses, balanced and unbalanced translocations and other structural aberrations as observed in patient-derived cell lines indicating commonality of alterations in cell lines and clinical samples together with disease heterogeneity [36].

Alterations observed in MF/SS patients were found to be present in the same chromosomal regions as in patient-derived cell lines. The most common chromosomal losses observed between the cell lines and MF/SS patient samples included 1p36.1, 9p21, 13q14 and 16q24 regions. The karyotype analysis of the cell lines and patient MF/SS samples revealed that structural changes can predict CTCL disease variant since loss at 10q24 region was observed in biospecimens from SS patients as well as in blood-derived cell lines including SeAx, Sez4, SZ4, H9, Hut78 and HH, but not in skin-derived MF (MyLa and HH) and CD30^+^ LPD (PB2B and Mac2A) cell lines [36]. Taken together, these findings suggest that 10q24 loss is a marker for a leukemic form of CTCL.

### 2.3. Gene Expression Profiling of Cell Lines and Patient Samples

Gene expression profiling is another method to decipher similarities in CTCL cell lines and patient samples. Previously, various gene expression studies have been conducted on MF/SS/CD30^+^ LPD cell lines and patient samples to identify novel diagnostic and prognostic markers [19,49,50,51,52]. In addition, the expression of oncogenes and tumor suppressor genes has been evaluated to determine their role in disease pathogenesis [53,54].

In the past, 107 commonly studied in CTCL genes were analyzed using transcription profiling in 11 cell lines using unsupervised clustering to assess similarities and differences between them. Analysis of naturally occurring patterns in transcriptomics data of cell lines differentiated them into two clusters. In the first cluster, cell lines were derived from advanced MF (MyLa and HH) and CD30^+^ LPD (PB2B and Mac2A) patients; within this cluster, Mac2A and PB2B clustered together as these cells were derived from the same patient at different stages and possessed considerable similarity at the genomic level. Notably, this similarity was also reflected in spectral karyotyping mentioned above [36].

Cluster 2 contained cell lines derived from SS patients, including Sez4, SZ4, SeAx, H9, and Hut78 and HTLV-1^+^ cell lines MJ and Hut102. The SS cell lines clustered in one cluster 2A while HTLV-1^+^ cell lines clustered in 2B separately from the other SS cell lines. Sez4 and SZ4 clustered together, as supported by the karyotyping analysis. These cell lines were found to be very similar to SeAx cells based on the gene expression analysis data. Hut78 and H9 clustered together based on expression profiling, as supported by the spectral karyotyping analysis. Hence, the gene expression studies also confirmed that MyLa and HH represent advanced MF; PB2B and Mac2A represent CD30^+^ LPD while Sez4, SZ4, SeAx, H9, and Hut78 represent SS. In contrast, MJ and Hut102 clustered together. Considering the aforementioned karyotyping analysis together with the unsupervised clustering results based on gene expression, these cells likely represent ATLL and not MF/SS [36].

Additional gene/protein expression analyses have been carried out in cell lines to identify markers that may be useful for disease diagnosis, prognostication, and treatment. As the disease progresses, tumors acquire a Th2 inflammatory profile and attenuate the Th1 inflammatory response. Litvinov et al., (2014) [18], evaluated the expression of Th1, Th2, Th9, Th17 and Treg markers in the CTCL cell lines. The expression of Th1 markers was either not observed or attenuated (only Hut78 expressed IFN-γ upon stimulation) but these cell lines heterogeneously expressed Th2 markers (e.g., IL-4). MyLa (advanced MF), Mac2A (CD30^+^ LPD), SeAx (SS), Hut78 (SS), H9 (SS), MJ (ATLL) and Hut102 (ATLL) expressed IL-4 in standard culturing conditions or upon stimulation with phorbol 12-myristate 13-acetate and ionomycin or with CD3/CD28 Dynabeads^®^. IL-17F, IL-17A or both were expressed by MyLa (advanced MF), PB2B (CD30^+^LPD), SZ4 (SS), Hut102 (ATLL), and SeAx (SS), which correlated with the known increased expression of IL-17F and IL-17A in MF patches and plaques [55]. Treg markers (e.g., TGF-β1) were variably expressed in MyLa (advanced MF), Mac2A (CD30^+^ LPDs), PB2B (CD30^+^ LPDs), SeAx (SS) and HH (advanced leukemic MF) cells [18]. In the 11 cell lines, downregulation of IFN-γ and SERPINB13 was observed. IFN-γ is essential for the attenuation of Th2 TME and stimulation of CD8^+^ T cells as well as NK T cells [56]. While SERPINB13 plays an essential role in the inhibition of cathepsin K, responsible for cancer cell invasion [57]. Thus, decreased levels of IFN-γ and SERPINB13 together with the decreased expression of Th1 inflammatory markers and increased expression of Th2, Th17, and Treg markers [36,58] signifies advanced disease stage of all patient-derived cell lines. The enhanced expression of CD30 (therapeutic target in CTCL) was especially observed in PB2B and Mac2A cells as these were harvested from a known CD30^+^ LPD patient.

The cell lines including Myla, SeAx and Mac2A have also been investigated for cell cycle and proliferation markers. The study revealed increased expression of cyclin E (predominately expressed in the S phase and decreased in G2/M phases in non-malignant cells) during S and G2/M phases in these cells. Proliferation marker PCNA (Proliferating cell nuclear antigen), a subunit of DNA Polymerase δ was found to be positive in 90% of cells regardless of growth rate. Thus, PCNA may not be a useful differentiating marker of proliferation in CTCL cell lines [59].

MF and SS can also be distinguished based on the expression of inflammatory marker, IL-10. IL-10 levels were undetectable in MF/CD30^+^ LPD cell lines including MyLa, HH, Mac2A and PB2B, but were observed in SS cell lines. In addition to these poor prognostic markers, other genes such as *ITK*, *AHI1*, *TRAF3IP*, *FYB*, *KIT*, *LCK* and *TBX3* were found to be upregulated in SS.

TP53 has been often reported to be altered in advanced disease CTCL patients [60]. The analysis was performed on the aforementioned cell lines and synonymous alteration at codon 72 was observed in MyLa (advanced MF), Mac2A (CD30^+^ LPD), PB2B (CD30^+^ LPDs), HH (advanced leukemic MF), SeAx (SS), SZ4 (SS), Sez4 (SS), Hut78 (SS), H9 (ATLL) and Hut102 (ATLL) cells. Nonsense mutations were noted in exon 6 of Hut78 and its clonally derived variant H9; impacting the functional property of the p53 protein. SeAx cells exhibited a deleterious alteration in exon 7. These findings are consistent with the previous reports in CTCL patient samples, therefore, indicating advanced disease represented by mutated *TP53* in Hut78, H9 and SeAx Sézary cells [36].

Immortalized cell lines are a powerful tool and offer several advantages over primary cells as they are renewable, easy to manage, and consistent to study between different laboratories. The immortalized cell lines do not fully represent primary cells and researchers have to be cognizant of possible inconsistencies that arise from genomic instability that contributes to variability with each passage number. Also, cell lines representing early stage CTCLs need to be developed. In part, to overcome these limitations and support studies on the TME, several xenograft/GEMMS have been developed to study CTCL.

## 3. Mouse Xenograft Models

Animal models are vital in studying and understanding the disease etiology, allowing researchers to test and optimize novel therapeutic strategies in vivo prior to clinical trials [61]. Previous studies report various attempts to model CTCL in mice. However, mice do not spontaneously develop CTCL or epitheliotropic T-cell lymphoma that is seen in other animals [62]. The first-ever CTCL study using an animal model was generated ~30 years ago [63,64]. A skin graft from a SS patient was placed in the lateral thorax area of the mouse [65]. Implantation of skin graft in that mouse model was followed by an intravenous and intraperitoneal infusion of many SS leukemic cells. After one month, despite the presence of patient graft and large number of leukemic cells, CTCL remained restricted to the skin graft only and no disease was seen in the peripheral blood or spleen of the model organism [65]. Because the success with these early engraftment models was highly variable, reproducibility was low and the platform was very laborious and expensive, these models were abandoned. In addition, they also failed to fully mimic human disease.

### 3.1. Xenograft Mouse Models Using Patient-Derived Cell Lines

The next attempt to establish a more convenient CTCL animal model was the pioneering work by Thaler et al., (2004) [66], who performed subcutaneous transplantation of MyLa MF cells in aplastic nude nu/nu mice [66]. In this study, initial tumors were established by injecting 10 × 10^6^ MyLa cells subcutaneously, followed by excision of tumors from mice after they reached a volume of 1.5 cm^3^. These tumors were sectioned and replanted in the flanks between hind and fore flank subcutaneously and within 40–60 days tumors of 1 cm^3^ in size were observed in 83% of mice (Table 2). Histochemical staining and PCR confirmed the spread of tumor cells in the lymph nodes, peripheral blood, liver, and lungs. This observation corresponded well with clinical observations in advanced MF patients. The limitation of this model was the need for repeated passages of the tumor cells to enable them to adapt for studies in nu/nu mice, with the risk of selecting tumor cells that were likely to deviate from the original malignant T cell line due to the inherent genomic instability of MyLa cells and the new selection pressures. Accordingly, the model never gained support or common use in CTCL research.

SS cell lines, Hut78 and SeAx, have been used as grafts in CB-17 SCID beige mice (CB-17/lcr.Cg-Prkdc scid Lyst bg/Crl) to evaluate the tumorigenic efficiency of cells. Specifically, 3 × 10^6^ cells were injected subcutaneously into the flank of mice. The tumor was observed only in Hut78 injected mice after 44–62 days, but no T cells were observed in the blood of mice, and no metastasis was noted. No tumor development was observed in mice injected with SeAx cells (Table 2) [67]. This study provided useful evidence that grafts from Hut78 but not SeAx can survive in this mouse model.

Contemporaneously, Krejsgaard et al., (2010) used the NOD.Cg-Prkdc ^scid^ B2m^tm1Unc^/J mouse model engrafted with MyLa2059 to study the development of CTCL [2]. Subcutaneous transplantation of the malignant T cells led to rapid tumor formation within 15 days in 90% of cases, whereas transplantation of non-malignant T cells isolated from the same donor did not result in tumor development. In most mice, the tumors displayed subcutaneous and/or lymphatic dissemination. Histological, immunohistochemical and flow cytometric analyses confirmed that both tumors at the inoculation site, as well as distant subcutaneous and lymphatic tumors, originated from the transplanted malignant T cells (Table 2). The model was the first to be used to evaluate treatment efficacy for CTCL xenograft. Treatment with the clinically approved HDAC inhibitor for CTCL, vorinostat (suberanilohydroxamic acid) supressed tumor growth as compared to mice treated with vehicle alone, demonstrating the applicability of this engraftment model [2].

MF cell line, HH was also engrafted in NOD/Shi-scid, IL-2Rgamma(null) (NOG) mice. The xenograft mouse model was used to evaluate responses to KM2760, a chimeric defucosylated anti-CCR4 monoclonal antibody (mAb). Prior to inoculation, NOG mice engrafted with the tumor were randomized into 4 groups. These groups were treated with KM2760, or KM2760 given with peripheral blood mononuclear cells (PBMCs), human PBMCs alone and normal saline (vehicle) respectively, to study the efficacy of the drug. It was observed that mice receiving PBMCs and KM2760 combination therapy had smaller tumors in comparison to the treatment with the vehicle, human PBMCs or KM2760 alone (Table 2). This study determined that the use of KM2760 in the presence of immune cells (i.e., human PBMCs) is more efficient in treating CTCL, when compared to the use of drug or PBMCs alone [70]. Thus, this xenograft mouse model helped evaluate the efficacy of KM2760, a predecessor of the currently approved mogamulizumab.

Wu et al., (2021) used NSG mice (NOD.Cg-Prkdcscid Il2rgtm1Wjl/SzJ) and injected 6.6 million cells from MF patient (stage IIB) into the mice flank subcutaneously. After 19 days, mice with cells from MF patient (P0, PRS-1-P0) developed erythematous patches, scaly skin lesions and alopecia. Lymphocyte infiltration was observed in the skin, spleen and liver. Atypical lymphocytes were detected in the epidermis. Analysis of spleen and affected skin from PRS-1-P0 mice confirmed that malignant cells expressed CD3 and CD4 without co-expression of CD7; these findings matched the CD antigen profile of the donor patient. In this study PBMCs from two SS patients (Stage IVB and IVA) were injected intravenously through a tail vein into the NSG mice (PRS-2-P0 and PRS-3-P0). These mice developed erythematous patches, scaly skin lesions and disseminated disease that involved spleen and visceral organs. Sézary cells were observed in the blood of the PRS-2-P0 mouse. CD profiling of the affected skin and spleen revealed the expression of CD4 without co-expression of CD7 in PRS-2-P0 mice. These patient derived xenograft (PDX)-harboring mice were used to evaluate the therapeutic efficiency of BKM120 compound, and it was found to inhibit cell growth of human malignant T cells in PRS-1 and PRS-3 but not PRS-2 mice. Further, experiments were carried on PRS-1 (MF PDX model). BKM120 treated PRS-1 showed reduced tumor burden represented by smaller spleens, reduced tumor growth measured by cfDNA and prolonged survival in comparison to control mice. The study established that the MF PDX model could be used to test BKM120, a compound targeting Phosphoinositide 3-kinase (PI3K) pathway [71].

Paglio et al., (2021) used NSG mice (NOD.Cg-Prkdc(scid)Il2rg(tm1Wjll)/SzJ) for in vivo modelling of SS Samples. PBMCs from 14 patients were injected intrafemorally into NSG mice. Only 14.3% (2 out of 14) (i.e., patients # 2 and 10) engrafted Sézary samples developed the disease. At the time of sacrifice, patient Sézary cells invaded different tissues such as femur, spleen, liver, kidneys, and blood. Skin lesions were observed in mice when secondary xenograft (i.e., cells derived from patient #2) was injected percutaneously, and local tumorigenesis was observed in 100% of animals. In this case, disease spread to different tissues was observed in mice. Such preclinical models could be used to analyze both epidermotropism and leukemic/metastatic spread of the disease [69].

Several additional studies have used xenograft mouse models to decipher the diverse role of various oncogenes in CTCL. In a study, Hut78 and HH cells were used to explore the role of TOX a prominent oncogene in CTCL via implantation of cells with or without knock-down of the gene [51]. These cells were injected subcutaneously in both flanks of NSG mouse models. The mice with xenografts characterized by higher TOX expression developed aggressive subcutaneous tumors, compared to the mice injected with Hut78 and HH cells where TOX was knocked down. These NSG mouse models highlighted the contribution of TOX to CTCL lymphomagenesis [72].

NSG-based xenograft mouse model has also been used to decipher the role of MUC1-C oncoprotein in CTCL. NSG mice were injected subcutaneously in the flanks with Hut78 cells. Previously, it has been reported that CTCL cells exhibit resistance to ROS-mediated apoptosis and one of the reasons for high ROS is increased levels of MUC1-C. Xenografts were injected with GO-203, a MUC1-C inhibitor, intradermally for 21 days after the tumor reached an average of 5–7 mm^3^ in size. On day 22, Hut78 bearing xenograft mice were analyzed for the presence of apoptotic cells via a TUNEL assay concomitantly with MUC1-C staining (Table 2). More apoptotic cells were present in GO-203 treated mice in comparison to the tumors isolated from non-treated mice, confirming the role of Mucin1 in attenuating apoptosis [68].

CTCL cell lines MyLa, Hut78, SeAx, or HH were also injected intrahepatically into the NSG mice (Table 2). It was observed that intrahepatic placement of these aggressive cells leads to the tumor formation in the liver of the hosts [73] although precise relevance to human disease of these models is unclear, given that this microenvironment is not reflective of tissues associated with CTCL.

Subcutaneous xenografts of Mac2A, PB2B, MyLa, Hut78, HH, H9, Sez4, SeAx, SZ4, MJ and Hut102 cells were implanted in the NOD.Cg-Prkdc^scid^ Il2rg^tm1Wjl^/SzJ (NSG) mice. Tumors were established by 8/11 cell lines when implanted subcutaneously. Mac2A, Sez4 and SeAx cells failed to produce tumors while PB2B produced small tumors. MyLa, HH, H9 and Hut78, produced most aggressive tumors within 2–4 days of implantation and SZ4 cells produced smaller tumors still at week 4 [36]. MJ and Hut102 produced tumors at weeks 8 and 9, respectively. In addition, expression of CD4, CD5, CD7, CD8, CD30 and CD45RO markers as well as T cell receptor (TCR) clonality was analyzed in the tumors. Expression of CD7 was found to be absent in all tumors excised. Expression variability was observed for CD3 and CD5 markers. Expression of CD5 was not observed in tumors excised from mice that had PB2B and MyLa engrafts. CD30 and CD45RO was strongly expressed in all tumors. Hence, this study gave valuable information on the expression of cell surface markers associated with CTCL and defined which of the known cell lines are able to engraft in NSG mice and establish tumors (Table 2).

Thus, xenograft models using CTCL cell lines in various immunodeficient mice have been a helpful tool to evaluate tumor development of malignant T cells in genetically defined hosts and, importantly, provided new means to study treatment efficacy in an in vivo setting. As the TME and the interplay with bacteria such as *Staphylococcus aureus* and other environmental factors are believed to play a key role in disease progression [78,79,80], xenograft models suffer from a number of limitations and shortcomings in relation to the lack of a relevant TME and interaction with host immune system, which stresses the need for alternative and novel mouse CTCL models, as discussed below.

### 3.2. GEMM and Other Important CTCL Mouse Models

Wild-type C56BL/6 mice is another mouse model for CTCL. Wild type C56BL/6 mice can be used as a host for murine MBL2 T lymphoma cells, an inflammation dependent CTCL model. It was observed that tumors developed after the application of a contact sensitizer di-nitro-fluoro-benzene (DNFB) immediately after the inoculation of MBL2 T cells into skin of host animals. This model can be used as a preclinical model to study the role of inflammatory modulators in CTCL. The model is largely inflammation dependent, and resolution of inflammation leads to the resolution of lymphoma. While BALB/c mice do not support implantation of MBL2 tumors, B6.SJL mice can be easily inoculated with MBL2 without underlying inflammatory microenvironment (Table 2) [74,81,82,83].

Mishra et al., (2016) evaluated the role of an inflammatory cytokine IL-15 in CTCL using IL-15 transgenic (Tg) mice that developed skin inflammation with some features reminiscent of human MF. Lymphocytes isolated from skin of IL-15 Tg CTCL mice were administrated in SCID mice. Approximately, 2 × 10^6^ cells inoculated subcutaneously into the right flank of each SCID mouse led to CTCL development. Using this model, the authors indicated that increased IL-15 leads to the spontaneous CTCL developed in SCID mice and the study concluded that excessive autocrine production of IL-15 inhibits negative autoregulatory loop for HDAC1, resulting in the upregulation of HDAC1 and HDAC6 and transcriptional induction of the onco-miR-21 [75]. These results highlight the role of IL-15 in CTCL pathogenesis and indicate that this transgenic model is an intriguing platform to study CTCL (Table 2). Furthermore, the disease can be established in a transplant model upon transfer of skin tropic T cells from IL-15 Tg mice into SCID hosts.

Adachi et al., (2015) used *Rag2*^−/−^ and transferred *Myc*^+^*Cdkn2a*^−/−^ CD4^+^ T cells into immune compromised host. After inoculation, the erythroderma was observed in *Rag2*^−/−^ as well as other histological features reminiscent of CTCL. Notably, in this murine model the CD4^+^ epidermotropsim was dependent on IL-7 and IL-15 stimulation, cytokines that are produced by hair follicles [76]. The role of these inflammatory cytokines has been previously established in the pathogenesis of CTCL and the transfer model further emphasized their role in CTCL pathogenesis [75]. These studies highlight the critical association between persistent inflammation and malignant transformation in CTCL (Table 2).

Fanok et al., (2018) used a *R26STAT3C^stopfl/+^ CD4Cre* mouse model to study the role of JAK/STAT signaling in T cell lymphomagenesis. In this model conditional gene targeting resulted in upregulation of STAT3 signaling exclusively in T cells. These mice were found to have significantly increased number of CD4^+^ T cells in secondary lymphoid organs and striking dermal tropism of T cells with many pathognomonic features of human CTCL present. Flow cytometric analysis revealed highly augmented IL-17 and IL-22 cytokine production by T cells in these mice compared to littermate controls [77]. These findings were consistent with the skin biopsy findings of some MF patients, where increased amounts of IL-17A were observed [18,55]. The study demonstrated the role of elevated STAT3 in potentiating the expression of inflammatory markers, which contributes to inflammatory skin environment in CTCL. Of notice, disease development was dramatically ameliorated in mice living under germ-free conditions, compared to mice housed under standard conditions. This finding suggests that microbiota plays an important role in fueling disease progression, further highlighting the potential of using genetically modified mouse models to study the role of TME in disease pathogenesis and progression [77,84] (Table 2).

Xenobiotic murine models offer a platform to investigate the etiology of CTCL. These models provide an excellent system that allows engraftment of cells from established CTCL cell lines or CTCL patient skin, providing tools to develop greater understanding of pathways involved in the pathogenesis of CTCL. Numerous genetic alterations have been reported to be a causative factor of CTCL. The causality of these driver mutations can be tested by using GEMMs and in turn these models provide invaluable tools for further interrogation of novel molecular drivers of disease and for dissection of the role of TME in disease pathogenesis [85]. The studies on murine models provide an insight into the causative role of interleukins in CTCL, the role of microbial drivers of inflammation and represent tractable preclinical platforms for novel therapeutics.

## 4. Conclusions

Cell lines and xenograft/GEMMs are valuable tools to study the etiology of CTCL. These models have been used to evaluate the therapeutic efficacy of various drugs prior to clinical trials. Although these are valuable models and are continually evolving to incorporate new technologies and biological advances, there is still a need for better representation of clinical disease and its variants. Notably, models that represent early stages of CTCL are lacking.

Novel murine models are also needed to study CTCL as one of the drawbacks of using NSG/SCID mice is that they don’t provide the same immune environment within the lesional skin, as observed in humans, and fail to consider interactions between lymphoma cells and host immune system. The genetic models highlighted in the review each have their own limitations as most recapitulate some, but not all features of the human disease and by their very nature are often driven by a singular signaling pathway with little room for establishment of additional genetic aberrations. To overcome this limitation murine models with humanized immune system that can be used as hosts for patient-derived tumor cells are likely to prove highly valuable for studying the pathogenesis of CTCL. Such models will encapsulate advantage of both xenobiotic transfer models (relevant primary malignant cells) and GEMMs with intact immune system that allow to explore TME and its role in disease development.

The current review highlights different cell lines and mouse models available for the study of CTCL. Hence, we hope that in each investigator selects the most appropriate model not merely based on availability but tailored to answer a scientific question at hand. Our review also strives to clarify the relationship between existing cell lines highlighting common origins of these frequently used cells.

## Figures and Tables

**Table 1:** Summary of the cell lines used to study CTCL.

Cell Line	Age, Race and Sex (Year Established)	Clinical Stage/ Tissue Source	Information on the Patients	Immunophenotyping Details	Media/Culture Requirements	Disease Representation	Reference
Cell lines representing MF							
MyLa	82, Caucasian male (1990)	IIA Skin Biopsy	Patient was diagnosed with MF based on histology of Pautrier microabscesses; presence of lymphadenopathy.	CD4^+^ and CD8^+^ forms	RPMI-1640 FBSPen/strep	Advanced MF	[27]
HH	61, Caucasian male (1986)	IVB Blood	Lymphadenopathy was observed in the patient.	CD2^+^, CD3^+^, CD4^+^, CD5^+^, CD30^+^, CD8^−^ and CD25^−^	RPMI-1640 FBSPen/strep	Advanced leukemic MF	[28]
Cell lines representing CD30^+^ LPDs							
Mac2A	47, Caucasian male (1987)	Ann Arbor stage ≥III Skin tumors	Patient initially diagnosed with Hodgkin’s disease, lymphomatoid papulosis (LyP) followed by cALCL. He then developed CTCL. Extensive CD30^+^ ALCL cells were detected in retroperitoneal lymph nodes, bilateral inguinal lymphadenopathy was observed. It was observed that Hodgkin disease, Lyp and CTCL/ALCL all originated from single T cell clone.	CD30^+^, CD15^+^, CD2^−^	RPMI-1640 FBSPen/strep	CD30^+^ LPD	[29]
PB2B	47, Caucasian male (1987–1988)	Ann Arbor stage ≥III Skin tumors	Bilateral inguinal lymphadenopathy was observed. The cell line was established from same patients from which Mac2A was established but it was established at a later aggressive stage.	CD30^+^, CD15^+^, CD2^−^	RPMI-1640 FBSPen/strep	CD30^+^ LPD	[29]
Cell lines representing SS							
SZ4	66, African-American female (1986)	IVA Blood	Lymphadenopathy in axillae, CTCL was diagnosed at stage IIA, advanced to stage IVA.	CD3^+^, CD4^+^, CD5^+^, CD25^+^, CD2^−^, CD8^−^, CD7^−^ and CD30^+^	RPMI-1640 FBSPen/strep	SS	[30]
Sez4	SZ4 and Sez4 cells were derived from the same patient. Inadvertently, at some point the name of this cell line in literature was changed from SZ4 to Sez4.				RPMI-1640 FBSPen/strep IL-2 and IL-4	SS	[30]
SeAx	66, Caucasian female * (1987)	IVA Blood	Patient suffered from severe exfoliative erythroderma with thick keratoderma of the palms and soles, nail dystrophy, hair loss, and lymphadenopathy, but no liver or spleen enlargement.	CD2^+^, CD3^+^, CD25^+^, CD4^+^, CD1^−^,CD5^−^, CD8^−^ and CD20^−^	RPMI-1640 FBSPen/strep IL-2 and IL-4	SS	[31]
Hut78	53, Caucasian male (1980)	IVA Blood	Skin, blood, lymph nodes and liver involvement were observed in the patient.	CD3^+^, CD4^+^, CD5^+^,	IMDM FBS Pen/strep	SS	[32]
H9	This cell lines is a clone of Hut78. The H9 clone was selected for permissiveness of HIV-1 replication, and has been used to isolate and propagate HIV-1 from the blood of patients with acquired immunodeficiency syndrome (AIDS) and pre-AIDS conditions. Established in 1983					SS	[33]
Cell lines representing ATLL							
Hut102	26, African-American male (1978)	IVA Lymph node	Skin lesions and lymph node involvement was observed. The cell line harbours HTLV-1 virus and TAX oncogene.	CD4^+^	RPMI-1640 FBSPen/strep	ATLL	[32,34]
MJ	50, Caucasian male (1982)	IVA Blood	The cell line harbours HTLV-1 virus and TAX oncogene.	CD4^+^	IMDM FBS Pen/strep	ATLL	[35]

MF: Mycosis Fungoides; RPMI-1640: Roswell Park Memorial Institute (RPMI) 1640; FBS: Fetal Bovine Serum; Pen/Strep: Penicillin/streptomycin; CD: Cluster of differentiation; IL: Interleukins; HTLV: Human T-lymphotropic virus; IMDM: Iscove’s Modified Dulbecco’s Medium; CTCL: Cutaneous T Cell Lymphoma; LPDs: Lymphoproliferative disorders; ATLL: Adult T-cell leukemia/lymphoma * Personal communication by Dr. Keld Kaltof.

**Table 2:** Detailed description of xenograft/GEMM mouse models used to study CTCL.

Mouse Model	CTCL Variant(s) Modelled	Observations	Study Design	Quality Score	References
C.B SCID mice	Graft from Sézary patient	CTCL remained restricted to the skin graft. Limitation: No disease was observed in peripheral blood or spleen.	SS leukemic cells were infused intravenously and intraperitonially in mice.	Poor disease representation	[65]
CB-17 SCID beige mice (CB-17/lcr.Cg-Prkdcscid Lyst bg/Crl) mice	SS cell lines: SeAx and Hut 78	Tumors were only observed in Hut-78-injected mice after 44–62 h. No T cells were observed in the blood and no metastases were noted. Limitation: No tumors were observed in mice injected with SeAx cells.	3 × 10^6^ cells were injected subcutaneously into the right flank of mice.	Fair disease representation	[66,67]
NSG mice	SS cell line, Hut-78	Tumors were analyzed for the presence of apoptotic cells; more apoptotic cells were observed in GO-203-treated mice in comparison to tumors isolated from non-treated mice. The study confirmed that MUC1-C attenuates apoptosis.	Hut78 cells were injected subcutaneously in the flanks of mice. Xenografts were injected with GO-203, a MUCI-C inhibitor for 21 days.	Acceptable disease representation	[68]
NSG mice (NOD.Cg-Prkdc(scid)Il2rg(tm1Wjll)/SzJ)	PBMCs from SS patients were engrafted	5 NSG mice for each patient were used. Tumors developed from 14.3% of engrafted samples. Skin lesions were observed in mice when secondary xenograft (i.e., patient-derived cells were injected percutaneously), and local tumorigenesis was observed in 100% of animals. Disease spread to different tissues in all mice. 8 new cell lines from four different patients were developed.	These models were established using patient cells. For the development of SC lines, spleen of mice were dissected and SC were sorted and plated in basal media with cytokines for 8 weeeks.	Acceptable	[69]
Aplastic nude nu/nu mice	Subcutaneous transplantation of advanced MF cell line, MyLa	Tumor cells were detected in lymph nodes, peripheral blood, liver and lungs of the model organism. Limitation: Repeated passages of tumor cells was needed to enable them to adapt for studies in nu/nu mice, risking the selection of tumor cells that were likely to deviate from the original MyLa cells due to repeated passages and selection pressure.	10 × 10^6^ cells were injected subcutaneously. Tumors were excised and subcutaneously replanted in the region between hind and fore flank.	Fair	[66,67]
NOD.Cg-Prkdc^scid^ B2m^tm1Unc^/J mice	Advanced MF cell line, MyLa 2059	Tumor were observed when MyLa2059 cells were transplanted subcutaneously. Tumors displayed subcutaneous and/or lymphatic dissemination. All tumors (subcutaneous site, distant subcutaneous and lymphatic tumors) originated from the transplanted T cells. Vorinostat supressed tumor growth in mice in comparison to the mice treated with vehicle alone.	The malignant T-cell line MyLa2059 and the non-malignant T-cell line MyLa1850 were obtained from a plaque biopsy of a patient diagnosed with MF. This model was used to evaluate the efficacy of HDAC inhibitor, Vorinostat.	Acceptable	[2]
NOG mice	Advanced MF cell line, HH	The HH bearing NOG mice were treated with KM2760 in combination with PBMCs responded better than the treatments with human PBMCs, KM2760 and vehicle alone. The use of KM2760 in the presence of immune cells had more pronounced effect in treating CTCL.	This model was used to evaluate the drug response for KM2760, a chimeric defucosylated anti CCR4 mAb, predecessor of currently approved mogamulizumab.	Acceptable	[70]
NSG mice (NOD.Cg-Prkdcscid Il2rgtm1Wjl/SzJ)	Xenografts from MF and SS patients	NSG mice with xenografts from MF patient showed lymphocyte infiltration in skin, spleen and liver. Atypical lymphocytes were observed in the epidermis. Analysis of spleens and affected skin confirmed that malignant cells expressed CD3 and CD4 without coexpression of CD7; these findings matched the CD antigen profile of the donor patient. PBMCs from two SS patients (Stage IVB and IVA) were injected intravenously through a tail vein into NSG mice. They developed erythematous patches, scaly skin lesions and disseminated disease that involved spleen and visceral organs. CD profiling of the affected skin and spleen revealed the expression of CD4 without coexpression of CD7. The study used a PI3K inhibitor, BKM120 and observed reduced tumor burden and tumor growth in mice treated with this compound in comparison to control mice.	6.6 × 10^6^ cells from MF patient and PBMCs from SS patients were used in the study.	Acceptable	[71,72]
NSG mice	SS cell line, Hut78 and advanced MF cell line, HH	Mice with active *TOX* gene developed aggressive tumors in comparison to the mice that received cells with *TOX* gene KD. This model confirmed to oncogenic properties of *TOX.*	Hut78 and HH cells with *TOX* gene KD vs. *TOX* active gene were injected subcutaneously in both flanks	Acceptable	[72]
NSG mice	Advanced MF cell lines: MyLa and HH SS cell lines: Hut-78, and SeAx	The study confirmed that intrahepatic placement of aggressive cells leads to the tumor formation intrahepatically in vivo.	Cells were injected intrahepatically into NSG mice.	Acceptable	[73]
NSG mice	Advanced MF cell lines: MyLa and HH CD30^+^ LPDs: Mac2A and PB2B SS cell lines: Hut78, H9, Sez4, SeAx and SZ4 ATLL cell lines: MJ and Hut102	Mac2A, Sez4 and SeAx failed to produce tumors. PB2B produced small tumors. MyLa, HH, H9 and Hut78 produced most aggressive tumors within 2–4 days. SZ4 produced smaller tumors at week 4. MJ and Hut78 produced tumors by weeks 8 and 9, respectively. TCR gene rearrangement was assessed. CD7 was a found to be absent in all tumors. CD30 and CD45RO was strongly expressed in all tumors. The study provided information on the markers expressed in CTCL and defined the cell lines that can establish tumors in NSG mice.	Subcutaneous xenografts were implanted in mice.	Acceptable	[36]
C56BL/6 mice	MBL2 cell line	The model can be used preclinically to test potential therapeutic agents and to study the role of inflammatory modulators in CTCL.	Mice were injected with MBL2 T lymphoma cells into the ear skin. Contact sensitizer, DNFB, to induce local inflammation was applied immediately after inoculation of MBL2 T cells.	Acceptable	[74]
B6.SJL mice	MBL2 cell line	The model can be used preclinically to test potential therapeutic agents and to study the role of inflammatory modulators in CTCL.	Subcutaneous implantation No need for inflammatory microenvironment (unlike in C57BL/6 mice) MBL2 cells are CD45.2; host cells are CD45.1	Acceptable	Dr. Neda Nikbakht personal communication
IL-15 transgenic mice	Not applicable	Spontaneous development of CTCL was observed due to increased levels of IL-15. Excessive autocrine production of IL-15 inhibits the negative autoregulatory loop for HDAC1, resulting in upregulation of HDAC1and HDAC6.	Skin cells taken from wild type mice were administrated into the IL-15 transgenic mice.	Acceptable	[75]
Rag2^−/−^ mice	*Myc*^+^*Cdkn2a*^−/−^ CD4^+^ T cells	Erythroderma and histological features indicative of CTCL were observed in mice. CD4^+^ epidermotropism was found to be dependent on IL-7 and IL-15 produced by hair follicles. The study established the role of inflammatory markers in CTCL.	*Myc*^+^*Cdkn2a*^−/−^ CD4^+^ T cells were transferred in the mice	Acceptable	[76]
*R26STAT3C ^stopfl/+^ CD4Cre* mice	Not applicable	High levels of IL-17 and IL-22 were observed in mice. The study demonstrated that STAT3 might be a causative factor for increased expression of inflammatory markers contributing to disease development.	A hyperactive version of STAT3, STAT3C, was knocked into the *Rosa26* locus with an upstream floxed stop cassette (*R26STAT3C^stopfl^*). Excision of the stop cassette mediated by Cre recombinase leads to expression of a flag-tagged STAT3C and concomitant expression of EGFP from the IRES-GFP cassette.	Acceptable	[77]

## Data Availability

All available data is presented in the paper.
